# Salidroside Protects Dopaminergic Neurons by Enhancing PINK1/Parkin-Mediated Mitophagy

**DOI:** 10.1155/2019/9341018

**Published:** 2019-09-10

**Authors:** Ruru Li, Jianzong Chen

**Affiliations:** ^1^Department of Neurology, The Air Force Hospital of Southern Theater Command, Guangzhou 510062, China; ^2^Department of Chinese Medicine, Xijing Hospital, Fourth Military Medical University, Xi'an 710032, China

## Abstract

Parkinson's disease (PD) is a common neurodegenerative disease characterized by the degeneration of nigrostriatal dopaminergic (DA) neurons. Our previous studies have suggested that salidroside (Sal) might play neuroprotective effects against PD by preserving mitochondrial Complex I activity. However, the exact mechanism of the neuroprotective effect of Sal remains unclear. Growing evidence indicates that PINK1/Parkin-mediated mitophagy is involved in the development of PD. In this study, we investigated whether Sal exerts a neuroprotective effect by modulating PINK1/Parkin-mediated mitophagy. Results showed that Sal alleviated MPTP-induced motor deficits in pole test. Moreover, Sal diminished MPTP-induced degeneration of nigrostriatal DA neurons as evidenced by upregulated TH-positive neurons in the substantia nigra, increased DAT expression, and high dopamine and metabolite levels in the striatum. Furthermore, in comparison with the MPP^+^/MPTP group, Sal considerably increased the mitophagosome and mitophagy flux. Moreover, in comparison with the MPP^+^/MPTP group, Sal evidently enhanced the mitochondrial expression of PINK1 and Parkin, accompanied by an increase in the colocalization of mitochondria with Parkin. However, transfection of MN9D cells with PINK1 siRNA reversed Sal-induced activated mitophagy and cytoprotective effect. In conclusion, Sal may confer neuroprotective effects by enhancing PINK1/Parkin-mediated mitophagy in MPP^+^/MPTP-induced PD models.

## 1. Introduction

Parkinson's disease (PD) is the most common movement disorder and the second most common neurodegenerative disease after Alzheimer's disease [1]. However, due to the clinical challenges of PD, including an inability to make a definitive diagnosis at the earliest stages of the disease, difficulties in the management of symptoms at later stages, and absence of treatments that slow the neurodegenerative process, no effective therapies to cure PD are available [2, 3]. Although the etiology of PD remains unclear, increasing evidence suggests mitochondrial dysfunction as a final common pathway in the pathogenesis of PD [4, 5].

Mitochondrial homeostasis is important for maintaining cell metabolism and function. Mitophagy is a key protective mechanism that selectively removes damaged or excessive mitochondria selectively via autophagy to maintain mitochondrial homeostasis [6, 7]. The role of mitophagy in PD was first highlighted from observed mitochondria marked by activated kinases within autophagosomes in neurons of PD patients [8]. Subsequently, an increasing number of evidences have underpinned the importance of mitophagy on the onset of PD [9–11]. However, the mechanisms that mediate impaired mitochondria for mitophagy are poorly understood. The studies in *Drosophila* for the first time depicted the effect of PINK1 and Parkin on mitochondrial function [12]. Today, numerous studies showed that PINK1/Parkin-dependent mitophagy has been identified potential targets for the treatment of PD [13–15]. Under resting conditions, PINK1 is constitutively imported into mitochondria and then rapidly cleaved and degraded. However, the degradation of PINK1 after import is disrupted when mitochondria are damaged, leading to PINK1 accumulation on the outer mitochondrial membrane and the recruitment of Parkin. Subsequently, Parkin ubiquitinates mitochondria and subsequently recruits ubiquitin-binding autophagy receptors, such as p62, to the mitochondria. Finally, damaged mitochondria are engulfed by LC3-positive phagophores and eventually fuse with lysosomes for degradation [16, 17].

Salidroside (Sal) is a bioactive component extracted from *Rhodiola rosea* L., which possesses multiple pharmacological properties, including antioxidant, antiaging, and antifatigue properties [18, 19]. Our previous studies have suggested that Sal may alleviate mitochondrial dysfunction by enhancing Complex I activity in MPP^+^/MPTP-induced PD models [20]. However, whether the neuroprotective effect of Sal is mediated by regulating mitophagy to alleviate mitochondrial dysfunction remains unknown. The present study was designed to (1) further assess the putative neuroprotective properties of Sal in an MPP^+^/MPTP-induced PD model and (2) determine whether the protective mechanisms involve modulating PINK1/Parkin-mediated mitophagy.

## 2. Materials and Methods

### 2.1. Cell Culture and Drug Treatments

MN9D cells were generated by the fusion of neuroblastoma with mice embryonic ventral mesencephalic cells [21]. This cell line is the closest to the primary mesencephalic dopaminergic (DA) neuron and commonly used as a DA neuron model to study PD [22]. MN9D cells were cultured in RPMI medium (HyClone Laboratories Inc., Logan, USA) with 10% FBS (Gibco, Gaithersburg, MD, USA) in a humidified atmosphere incubator of 5% CO_2_ at 37°C. The cells were pretreated with Sal (10, 25, and 50 *μ*M) for 24 h and incubated with 200 *μ*M of MPP^+^ for an additional 24 h based on a previous dose-effect study [20].

### 2.2. Cell Transfection

SiRNA duplexes were synthesized and purified by Biomics Biotechnologies Co. Ltd. (Nantong, China). MN9D cells were transfected with PINK1 siRNA (the sequences of si-PINK1 are depicted in Table 1) or scramble control siRNA using Lipofectamine 2000 (Thermo Fisher Scientific, Waltham, MA, USA) following the manufacturer's protocol. After 72 h, the transfection efficiency was detected by Western blot.

### 2.3. Animals and Drug Treatments

Adult male C57BL/6 mice (22–25 g) were purchased from the Fourth Military Medical University and were housed in a controlled environment (12 h on/off light cycle at 23 ± 1°C). According to a previous study, mice were randomly assigned to four groups (10 mice per group): Group A, control mice; Group B, Sal only (50 mg/kg/day); Group C, MPTP challenged; and Group D, MPTP (30 mg/kg/day)+Sal (50 mg/kg/day) [20]. The MPTP group received intraperitoneal injections with MPTP for five consecutive days (30 mg/kg/day) as previously used, and the control group and Sal alone-treated group received equal volumes of saline for five days. After an MPTP injection, mice of Groups B and D were administered with Sal (50 mg/kg/day) intraperitoneally for seven days, and the control and MPTP groups received an equivalent volume of saline. Mice were subjected to behavioral tests seven days after the last administration of MPTP or saline.

### 2.4. Behavioral Tests

The pole test was performed to evaluate the movement disorder caused by striatal dopamine depletion in PD models [23]. Briefly, mice were placed with their head facing upside on top of a rough-surfaced pole (8 mm in diameter and 50 cm in height). The time required for the mice to turn completely downward (T-turn) and climb down to the floor (T-LA) was recorded.

### 2.5. Immunofluorescent Assay

For an immunofluorescent assay, the cells were seeded onto chamber slides and fixed with 4% paraformaldehyde for 30 min and the animals were transcardially perfused with 4% paraformaldehyde. Brains were isolated, frozen, and cut into 30 *μ*m slices on a cryostat (Thermo Fisher Scientific, Waltham, MA, USA). Then, the sections and cells were permeabilized with 0.1% Triton X-100 for 30 min and blocked in 2% BSA for 30 min at room temperature. After washing with PBS, the slides and cells were incubated with antibodies against TH (Sigma-Aldrich, USA), DAT (Bioss, China), LC3B (CST, USA), and Parkin (CST, USA) at 4°C overnight and incubated with Cy3-labeled or FITC-labeled goat anti-rabbit IgG (Beyotime, Beijing, China) for 1 h in the dark. Mitochondria and nucleus were labeled with MitoTracker and DAPI, respectively. Images were investigated by using an inverted microscope (IX51-12PH, Olympus) and analyzed using ImageJ software.

### 2.6. High-Performance Liquid Chromatography (HPLC)

The dopamine, 3,4-dihydroxyphenylacetic acid (DOPAC), and homovanillic acid (HVA) levels in the striatum were measured by HPLC with electrochemical detection. Briefly, after the mice were sacrificed, we rapidly dissected the striatum and the neurotransmitters were extracted in 100 *μ*L of ice-cold perchloric acid via sonication. Homogenates were centrifuged at 13,200 × g for 10 min at 4°C. Supernatants were filtered (pore size: 0.45 *μ*m; Millipore, MA, USA) and then injected directly into the HPLC system. The dopamine, DOPAC, and HVA contents were determined with electrochemical detection (Eicom Corp., Kyoto, Japan). Concentrations of dopamine and its metabolites were expressed as ng/mg tissue weight.

### 2.7. Transmission Electron Microscopy (TEM)

For TEM, the cells or tissues were fixed in 2.5% glutaraldehyde for 4 h at 4°C. Then, the pellets were postfixed in 1% osmium tetroxide/0.1 M phosphate buffer (pH = 7.4) and dehydrated serial dilutions in acetone and embedded with the SPI-PON 812 Epoxy Resin Monomer (SPI Supplies Division, Structure Probe Inc., West Chester, PA). Ultrathin sections (60–80 nm) were stained with uranyl acetate and lead citrate and observed with TEM (Hitachi, Tokyo, Japan).

### 2.8. Western Blot

The total proteins of cells or tissues were collected using RIPA lysis buffer (Beyotime, Beijing, China), and the cytoplasmic and mitochondrial proteins in cells or tissues were collected using a Cytoplasmic Protein Extraction Kit and Cell or Tissue Mitochondria Isolation Kit (Beyotime, Beijing, China). Protein of equal quality was electrophoresed on an SDS-PAGE gel and transferred to PVDF membranes. The membranes were blocked and incubated with antibodies against LC3B (CST, USA), p62 (CST, USA), Parkin (CST, USA), PINK1 (CST, USA), TH (Sigma-Aldrich, USA), DAT (Bioss, China), and LAMP2A (Abcam, USA) antibodies at 4°C overnight followed by goat anti-rabbit IgG antibody (Santa Cruz Biotechnology Inc., USA). The membrane was visualized using chemiluminescent reagents and quantified using ImageJ software. All protein levels were adjusted for the corresponding *β*-actin (CST, USA) and VDAC1 (CST, USA) and were consistent across different treatment conditions.

### 2.9. Cell Viability

The cell viability was measured with MTT assay kit (Sigma-Aldrich, USA) following the manufacturer's instructions. The absorbance at 570 nm was measured and cell viability was expressed as the percentage to the control group.

### 2.10. Statistical Analysis

All experiments were repeated at least three times. The data were expressed as mean ± standard deviation. One-way analysis of variance and Tukey's multiple comparison test were used to analyze the results. A value of *P* < 0.05 was considered significant.

## 3. Results

### 3.1. Effect of MPP^+^ on Autophagic Flux

Our previous study showed that 200 *μ*M of MPP^+^ remarkably reduced the cell viability, and Sal (10, 25, and 50 *μ*M) markedly prevented the cell toxicity induced by MPP^+^ [20]. We first treated MN9D cells with 200 *μ*M of MPP^+^ for 0, 6, 12, 24, 36, and 48 h to investigate the level of mitochondrial autophagic flux in the MPP^+^-induced PD model at different time points. Western blot results indicated that after MPP^+^ treatment, the ratio of LC3II/LC3I peaked at 24 h and decreased over time. The expression of p62 was minimized at 24 h and increased over time ([Supplementary-material supplementary-material-1]; *P* < 0.05, *P* < 0.01). These results indicated that after MPP^+^ treatment, the autophagic flux of mitochondria was induced at an early stage and was damaged over time. Therefore, we chose the time point of 24 h for the following experiments.

### 3.2. Effect of Sal on MPTP-Induced Behavioral Impairment

**Figure 1:**
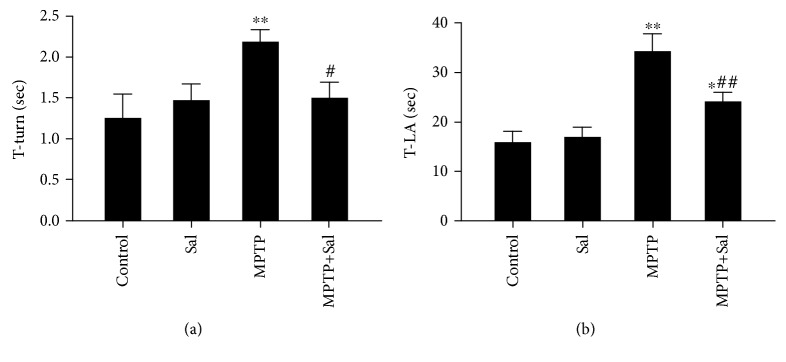
Effect of Sal on MPTP-induced behavioral impairment. The time obtained for mice to turn completely downward (T-turn) (a) and the time obtained for mice to climb to the floor (T-LA) (b) were determined using the pole test. Each column represents the mean ± SD (*n* = 10). ^∗^*P* < 0.05 and ^∗∗^*P* < 0.01, compared with the control group; ^#^*P* < 0.05 and ^##^*P* < 0.01, compared with MPTP-treated group.

Given that motor impairment is the main clinical backbone of PD, we assayed the behavioral deficits by using pole test as in the previous study [20]. The results of the pole test are shown in Figure 1. In the pole test, the time obtained to turn completely downward (T-turn) and the time obtained to climb to the floor (T-LA) are longer in MPTP-treated mice than in control mice (Figures 1(a) and 1(b); *P* < 0.01). Sal treatment significantly alleviated these behavioral disorders induced by MPTP (*P* < 0.05; *P* < 0.01), while Sal alone had no apparent effect.

### 3.3. Effect of Sal on MPTP-Induced DA Neuron Damage

**Figure 2:**
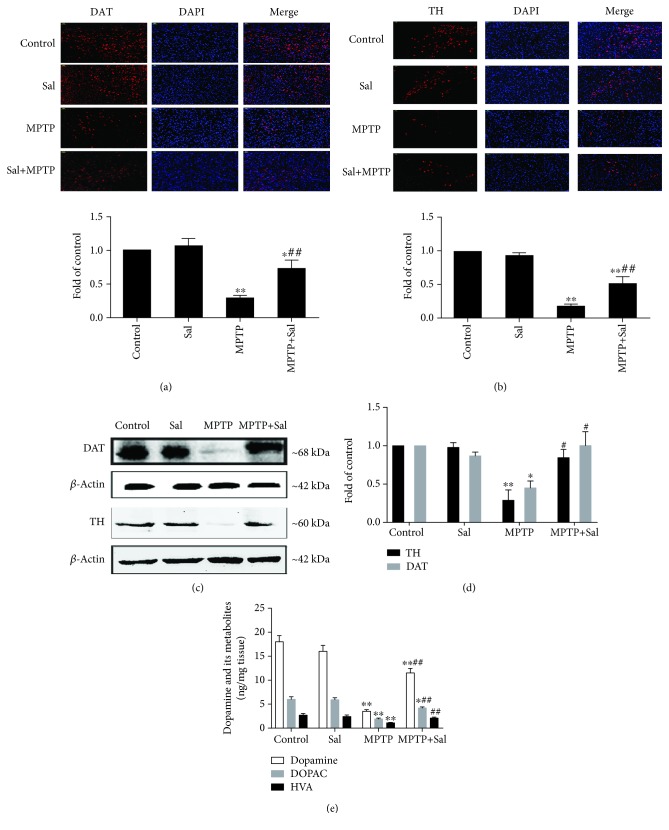
Effect of Sal on MPTP-induced DA neuron damage. Representative photomicrographs and quantitative analysis of DAT-positive neurons in the striatum (a) and TH-positive neurons in the SN (b). Scale bar, 50 *μ*m. (c) Western blot analysis of DAT expression in the striatum and TH expression in the SN of mice. *β*-Actin served as loading controls. (d) Qualification analysis of protein expression of DAT and TH in mice. (e) Effect of Sal on the level of dopamine, DOPAC, and HVA in the striatum of mice. Each column represents the mean ± SD (*n* = 3). ^∗^*P* < 0.05 and ^∗∗^*P* < 0.01, compared with the control group; ^#^*P* < 0.05 and ^##^*P* < 0.01, compared with the MPTP-treated group.

Given that nigrostriatal DA neurodegeneration correlates with the Parkinsonian motor features, we explored whether Sal ameliorated MPTP-induced DA neuron damage. Immunofluorescent assay results showed that Sal abrogated MPTP-induced decreases in TH-positive neurons in the substantia nigra (SN) and DAT-positive neurons in the striatum (Figures 2(a) and 2(b); *P* < 0.05; *P* < 0.01). Consistent with these results, Western blot results indicated that Sal ameliorated MPTP-induced decline in the protein expression of TH and DAT (Figures 2(c) and 2(d); *P* < 0.05; *P* < 0.01). Furthermore, HPLC results demonstrated that Sal reversed MPTP-induced reduction of dopamine, HVA, and DOPAC levels in the striatum (Figure 2(e)). Sal per se had no effect on DA neuron damage compared with the control group.

### 3.4. Sal Promotes Mitophagy in MPP^+^/MPTP-Induced PD Model

**Figure 3:**
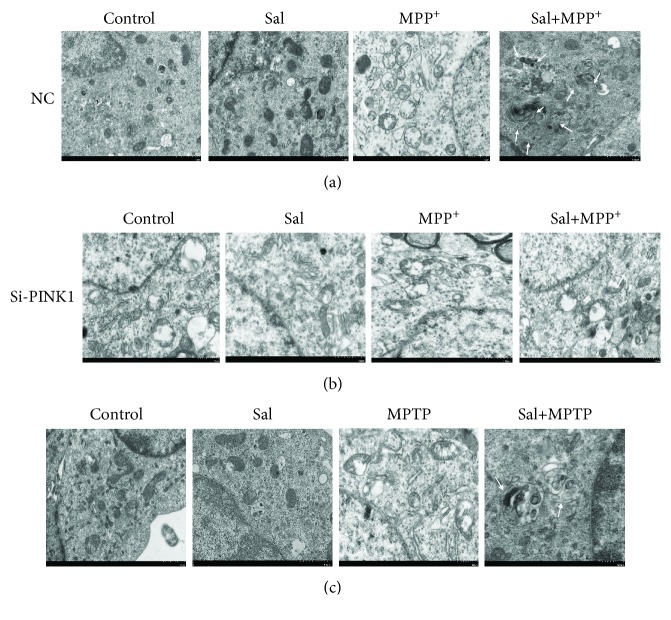
Sal treatment enhanced mitophagy in the MPP^+^/MPTP-induced PD model, and silencing PINK1 inhibited Sal-induced mitophagy. Representative TEM image of mitophagy autophagosomes in MN9D cells (a) and in MN9D cells transfecting with si-PINK1 (b). (c) Representative TEM image of mitophagy autophagosomes in the SN of mice. The arrows indicated mitophagy autophagosomes. Scale bar, 1 *μ*m.

**Figure 4:**
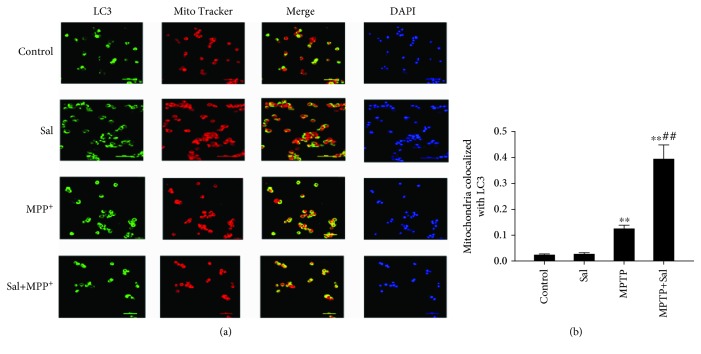
Sal pretreatment increased the colocalization of LC3 with MitoTracker. (a) Immunofluorescence colocalization analysis of LC3 with MitoTracker. (b) Quantification of Pearson's colocalization coefficient between LC3 and MitoTracker. Scale bar, 50 *μ*m. Each column represents the mean ± SD (*n* = 3). ^∗^*P* < 0.05 and ^∗∗^*P* < 0.01, compared with the control group; ^#^*P* < 0.05 and ^##^*P* < 0.01, compared with the MPP^+^-treated group.

**Figure 5:**
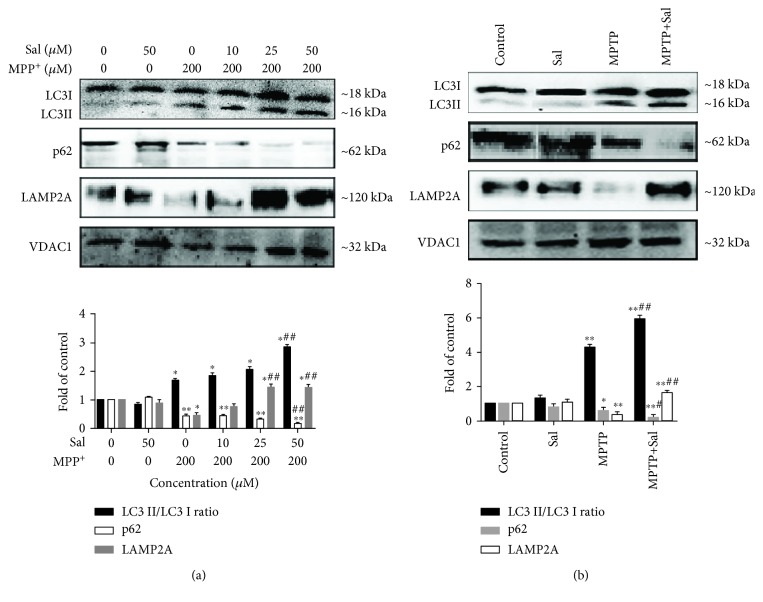
Regulation of LC3, p62, and LAMP2A expression. Regulation of the ratio of LC3II/LC3I, p62, and LAMP2A expression in the mitochondria of MN9D cells (a) and the SN of mice (b). Each column represents the mean ± SD (*n* = 3). ^∗^*P* < 0.05 and ^∗∗^*P* < 0.01, compared with the control group; ^#^*P* < 0.05 and ^##^*P* < 0.01, compared with the MPP^+^/MPTP-treated group.

Mitophagy is a form of selective autophagy that removes damaged or dysfunctional mitochondria to maintain cellular homeostasis and cell survival [6]. We examined whether Sal exerts a neuroprotective effect by inducing mitophagy. TEM results *in vitro* demonstrated that in comparison with the control group, MPP^+^ treatment increased the number of mitophagy autophagosomes, accompanied by elevated mitochondria damage. However, Sal pretreatment significantly induced more mitophagy autophagosomes and less mitochondrial damage in comparison with the MPP^+^ group (Figure 3(a)). Similar to the *in vitro* results, the *in vivo* results showed that Sal treatment significantly induced mitophagy in comparison with the MPTP group (Figure 3(c)). In addition, we further examined the formation of the “mitophagosome” by measuring the colocalization of LC3 and MitoTracker. Immunofluorescent assay results indicated that Sal pretreatment significantly increased the colocalization of LC3 and MitoTracker compared with the MPP^+^ group (Figure 4; *P* < 0.01). We also found that Sal significantly increased the mitochondrial ratio of LC3II/LC3I in comparison with the MPP^+^/MPTP group (Figure 5; *P* < 0.05; *P* < 0.01). Given that p62 is a marker of autophagy flux, we examined the mitochondrial protein expression of p62 by using Western blot. Compared with the MPP^+^/MPTP group, Sal significantly decreased the expression of p62 (Figure 5; *P* < 0.05; *P* < 0.01). We then measured the protein expression of lysosome protein LAMP2A, which is related to autophagy flux [24]. Western blot data indicated that Sal significantly induced the expression of LAMP2A in comparison with the MPP^+^/MPTP group. Sal per se had no apparent effect on mitophagy.

### 3.5. Sal Stimulates Mitophagy by Modulating the PINK1/Parkin Pathway

**Figure 6:**
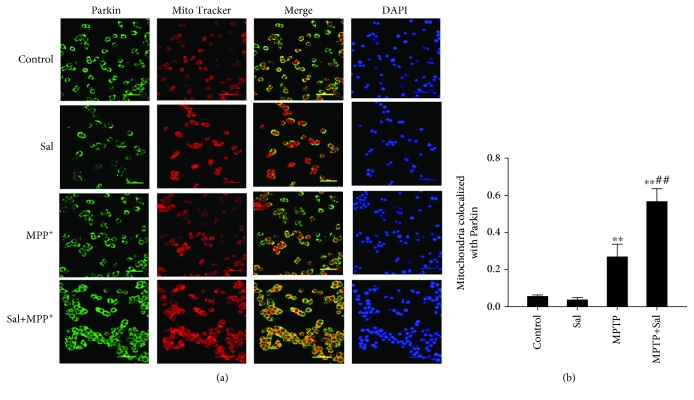
Sal pretreatment increased the colocalization of Parkin with MitoTracker. (a) Immunofluorescence colocalization analysis of Parkin with MitoTracker. (b) Quantification of Pearson's colocalization coefficient between Parkin and MitoTracker. Scale bar, 50 *μ*m. Each column represents the mean ± SD (*n* = 3). ^∗^*P* < 0.05 and ^∗∗^*P* < 0.01, compared with the control group; ^#^*P* < 0.05 and ^##^*P* < 0.01, compared with the MPP^+^-treated group.

**Figure 7:**
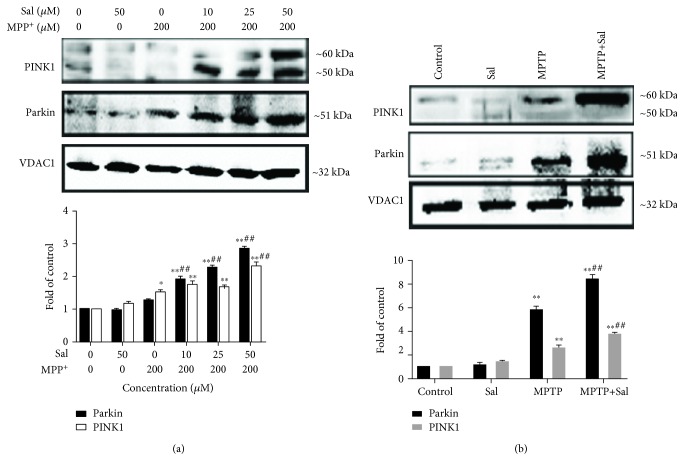
Regulation of PINK1 and Parkin expression. Regulation of PINK1 and Parkin expression in the mitochondria of MN9D cells (a) and the SN of mice (b). VDAC1 served as loading controls. Each column represents the mean ± SD (*n* = 3). ^∗^*P* < 0.05 and ^∗∗^*P* < 0.01, compared with the control group; ^#^*P* < 0.05 and ^##^*P* < 0.01, compared with the MPP^+^/MPTP-treated group.

**Figure 8:**
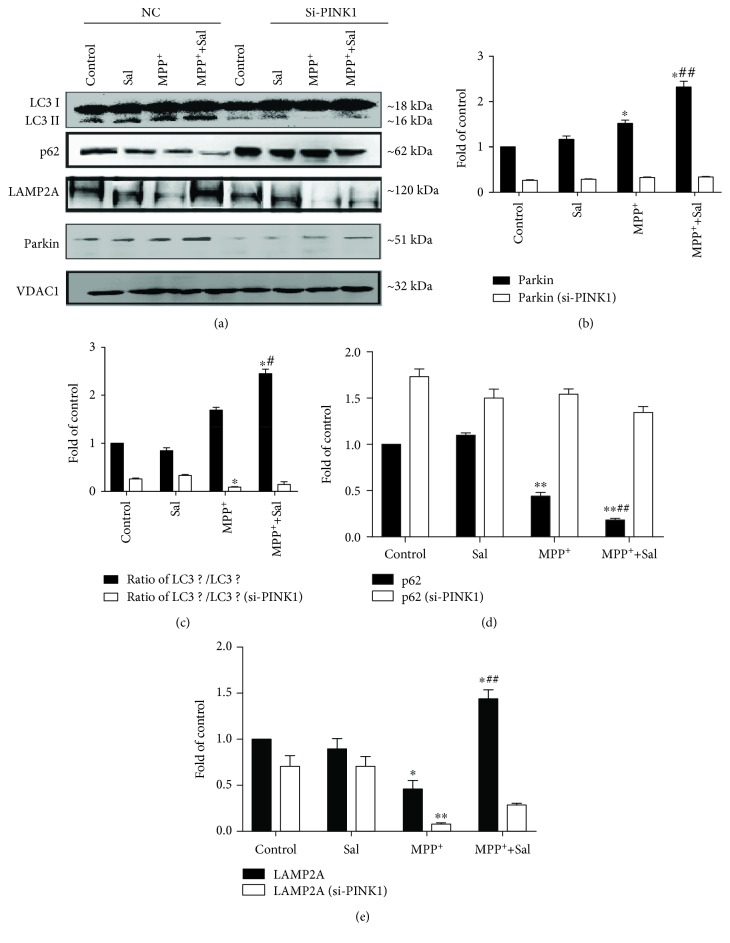
Silencing PINK1 inhibited Sal-induced activation of mitophagy in MN9D cells. (a) Mitochondrial protein expression of Parkin, LC3, p62, and LAMP2A in MN9D cells. VDAC1 served as loading controls. Qualification analysis of protein expression of Parkin (b), ratio of LC3II/LC3I (c), p62 (d), and LAMP2A (e) in the mitochondria. Each column represents the mean ± SD (*n* = 3). ^∗^*P* < 0.05 and ^∗∗^*P* < 0.01, compared with the control group; ^#^*P* < 0.05 and ^##^*P* < 0.01, compared with MPP^+^-treated group.

**Figure 9:**
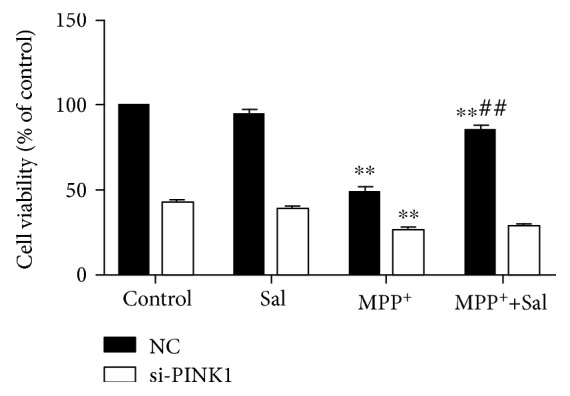
Silencing PINK1 inhibited Sal-induced activation of the cytoprotective effect in MN9D cells. Each column represents the mean ± SD (*n* = 3). ^∗∗^*P* < 0.01, compared with the control group; ^##^*P* < 0.01, compared with the MPP^+^-treated group.

Increasing evidence has suggested that the PINK1/Parkin pathway is involved in mitophagy [25]. Given that the translocation of Parkin to mitochondria is a hallmark of PINK1/Parkin-mediated mitophagy, we examined the colocalization of Parkin and MitoTracker [26]. Immunofluorescent assay results showed that Sal pretreatment significantly increased the colocalization of Parkin and MitoTracker in comparison with the MPP^+^ group (Figure 6; *P* < 0.01). Similar to immunofluorescent assay results, Western blot showed that Sal evidently increased the mitochondrial Parkin expression in comparison with the MPP^+^/MPTP group (Figure 7; *P* < 0.01). In addition, Sal significantly increased the mitochondrial expression of PINK1 in comparison with the MPP^+^/MPTP group (Figure 7; *P* < 0.01). We used siRNA to silence PINK1 expression in MN9D cells to further determine the role of PINK1/Parkin on Sal-induced mitophagy and neuroprotective effect. As shown in [Supplementary-material supplementary-material-1], si-Parkin (#2, 100 nM) was used for the following experiments. The TEM results showed that silencing PINK1 inhibited Sal-induced mitophagy in MN9D cells (Figure 3(c)). In addition, Western blot results showed that silencing PINK1 inhibited Sal-induced increase in autophagosome and autophagy flux as evidenced by the decrease in the LC3II/LC3I ratio and LAMP2A expression and the increase in p62 expression (Figure 8; *P* < 0.05; *P* < 0.01). Furthermore, MTT results showed that silencing PINK1 abrogated Sal-induced cytoprotective effect (Figure 9; *P* < 0.01). These findings support the role of PINK1/Parkin-mediated mitophagy in Sal-induced neuroprotective effect.

## 4. Discussion

In the present study, we demonstrated that Sal may play a neuroprotective effect by modulating PINK1/Parkin-mediated mitophagy as evidenced by the following: (1) Sal treatment ameliorated MPTP-induced behavioral impairment; (2) Sal treatment attenuated MPTP-induced DA neuron damage; (3) Sal treatment notably increased mitophagy; (4) Sal treatment activated the PINK1/Parkin pathway; and (5) silencing PINK1 inhibited Sal-induced mitophagy and cytoprotective effect.

The main symptoms of PD are motor disorders, such as muscle stiffness, tremor, bradykinesia, and postural instability, which are believed to be correlated with DA neuron loss. In the present study, we found that Sal alleviated MPTP-induced motor disorders as evidenced by the obvious shortened time for T-turn and T-LA in the pole test. The densities of TH and DAT are biomarkers for detecting the integrity of DA neurons. In the present study, Sal also diminished MPTP-induced decrease in TH-positive neurons in the SN and DAT-positive neurons, dopamine, and its metabolites in the striatum. Overall, our results showed that Sal markedly alleviated MPTP-induced DA neuron damage to recover motor function.

Our previous study had demonstrated that 200 *μ*M of MPP^+^ significantly decreases the cell viability of MN9D cells after 24 h exposure [20]. In the present study, we assessed the level of mitochondrial autophagic flux in MN9D cells treated with 200 *μ*M of MPP^+^ at different incubation times by measuring the ratio of LC3II/LC3I and p62 expression in mitochondria. Results showed that autophagic flux of mitochondria was activated by MPP^+^, peaking at 24 h and then decreasing thereafter. Thus, 200 *μ*M of MPP^+^ for 24 h was used for constructing *in vitro* mitophagy models.

Mitophagy is a specialized form of autophagy that removes damaged or excess mitochondria, thereby maintaining cellular function [6]. Growing evidence suggests deficits in mitophagy as a key mechanism in PD pathogenesis [7]. Targeting mitophagy may confer advantages of mitochondrial homeostasis and neuronal survival. In the present study, we observed that Sal treatment markedly increased mitophagy autophagosomes in comparison with the MPP^+^/MPTP group, as evidenced by the increase in autophagosome/autolysosome-engulfed mitochondria in TEM. LC3 colocalization with mitochondrial markers indicated that the damaged mitochondria are destined for autophagic degradation. In our study, we also observed the induced colocalization of mitochondria with endogenous LC3 after pretreatment with Sal in MPP^+^-induced MN9D cells. Upregulation of LC3II/LC3I concurrent with a decline in p62 is a biomarker of autophagy flux [27]. We also found that in comparison with the MPP^+^/MPTP group, Sal treatment increased the ratio of LC3II/LC3I and decreased p62 expression in mitochondrial fraction, suggesting that mitochondrial autophagic flux is activated in response to Sal. We further investigated autophagosome flux to the lysosome by measuring the expression of LAMP2A, a lysosomal transmembrane protein [24, 28]. Results indicated that Sal enhanced the protein expression of LAMP2A in comparison with the MPP^+^/MPTP group. On the basis of the aforementioned results, Sal may enhance mitophagy in the MPP^+^/MPTP-induced PD model.

The PINK1/Parkin pathway is the major mechanism underlying mitophagy. Increasing evidence suggests that the PINK1/Parkin pathway plays an important role in PD pathogenesis [13, 16]. The lack of PINK1/Parkin in SH-SY5Y cells disrupted mitophagy at different pathways [29]. PINK1 and Parkin deficiency in the midbrain of mice leads to a loss of DA neurons in the SN and a decline in mitochondrial mass [30, 31]. This finding is supported by our results in the present study, showing that Sal increased the colocalization of mitochondria with endogenous Parkin and the expression of PINK1 and Parkin in mitochondrial fraction compared with the MPP^+^/MPTP group. However, the transfection of cells with PINK1 siRNA reversed the Sal-induced increase in mitophagy autophagosomes, mitophagy flux, and cell viability in the MPP^+^-induced PD model.

However, our study has limitations. Our previous studies revealed that Sal treatment can preserve Complex I activity via the DJ-1/Nrf2 pathway to protect DA neurons against MPP^+^/MPTP [20]. Data show that lack of DJ-1 results in an aberrant mitophagy response in cells and in the SN of rats [32, 33]. Moreover, studies have indicated that activating Nrf2 can upregulate mitophagy in *Caenorhabditis elegans* and MEFs [34, 35]. Therefore, whether Sal exerts neuroprotective effects by activating mitophagy via the DJ-1/Nrf2 pathway is worthy of further study. Another limitation of our study was related to Sal. Sal is the main active bioactive component of *Rhodiola rosea* [19]. Once absorbed, Sal is subjected to extensive metabolism by phase I or phase II enzymes to undergo deglycosylation and might be further metabolized to its sulfate or glucuronide conjugates or even to methylate [36–38]. Thus, more studies need to be conducted to investigate the effect of possible physiological metabolites, such as tyrosol and phase II derivatives, on the PD models. Collectively, our study revealed that Sal could induce mitophagy through the PINK/Parkin pathway. The neuroprotective effects of Sal indicate its potential as a preventive therapy of PD.

## Figures and Tables

**Table 1:** The siRNA sequences of PINK1 in MN9D cells.

Gene	Forward primer	Reverse primer
*PINK1* (#1)	5′-UGGAUUUGUACCAUUCUUCUGdTdT-3′	5′-GAAGAAUGGUACAAAUCCAAGdTdT-3′
*PINK1* (#2)	5′-ACUCAUUGGUUCCUUUAAGGGdTdT-3′	5′-CUUAAAGGAACCAAUGAGUCCdTdT-3′
*PINK1* (#3)	5′-AGAAGUUUCGUUGAUAACCUGdTdT-3′	5′-GGUUAUCAACGAAACUUCUCAdTdT-3′

## Data Availability

The data used to support the findings of this study are available from the corresponding author upon request.

## Supplementary Materials

Supplementary Materials Figure S1: effect of MPP^+^ on the autophagic flux in the mitochondria of MN9D cells. (A) Western blot of the LC3II/LC3I ratio and (B) p62 expression in the mitochondria of MN9D cells treated with 200 *μ*M of MPP^+^ for the indicated time points. VDAC1 served as loading controls. Each column represents the mean ± SD (*n* = 3). ^∗^*P* < 0.05 and ^∗∗^*P* < 0.01, compared with the control group. Figure S2: protein expression of PINK1 after transfecting with various concentrations of siRNA for 72 h. Si-PINK1 (#2, 100 nM) was used for the following experiments. Each column represents the mean ± SD (*n* = 3). ^∗∗^*P* < 0.01, compared with the control group.
