# Supplementary material for: Salidroside Protects Dopaminergic Neurons by Enhancing PINK1/Parkin-Mediated Mitophagy
Source: Oxid Med Cell Longev. 2019 Sep 10;2019:9341018. doi: 10.1155/2019/9341018 (PMC6754964; doi:10.1155/2019/9341018)
Supplement: Supplementary Materials — Figure S1: effect of MPP+ on the autophagic flux in the mitochondria of MN9D cells. (A) Western blot of the LC3II/LC3I ratio and (B) p62 expression in the mitochondria of MN9D cells treated with 200 μM of MPP+ for the indicated time points. VDAC1 served as loading controls. Each column represents the mean ± SD (n = 3). ∗P < 0.05 and ∗∗P < 0.01, compared with the control group. Figure S2: protein expression of PINK1 after transfecting with various concentrations of siRNA for 72 h. Si-PINK1 (#2, 100 nM) was used for the following experiments. Each column represents the mean ± SD (n = 3). ∗∗P < 0.01, compared with the control group. [file 9341018.f1.pdf]

Supplementary Materials

Figure S1

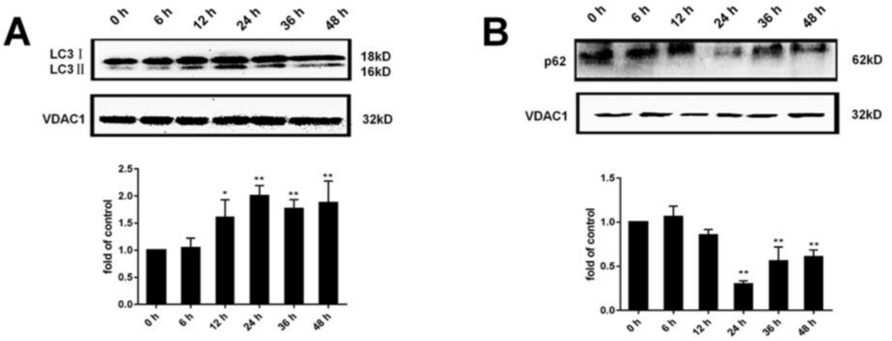

Figure S1 Effect of MPP^+ on the autophagic flux in the mitochondria of MN9D cells. **(A)** Western blot of the LC3Ⅱ/LC3Ⅰ ratio and **(B)** p62 expression in the mitochondria of MN9D cells treated with 200 μM of MPP^+ for the indicated time points. VDAC1 served as loading controls. Each column represents the mean ± SD (n=3). *P < 0.05 and **P < 0.01, compared with the control group.

Figure S1

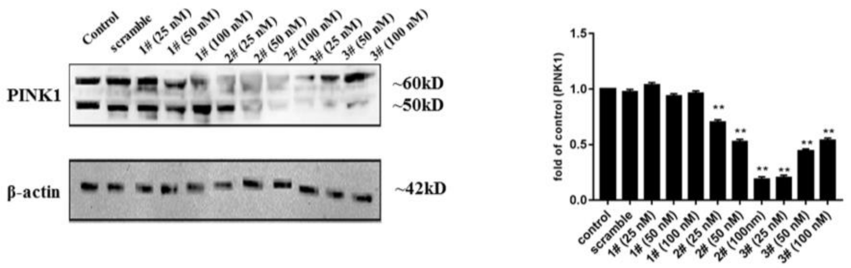

Figure S2 Protein expression of PINK1 after transfecting with various concentration of siRNA for 72 h. Si-PINK1 (**2#, 100 nM**) was used for the following experiments. Each column represents the mean ± SD (n=3). **P< 0.01, compared with the control group.
